# Influence of perioperative oxygen fraction on pulmonary function after abdominal surgery: a randomized controlled trial

**DOI:** 10.1186/1756-0500-5-383

**Published:** 2012-07-28

**Authors:** Anne K Staehr, Christian S Meyhoff, Steen W Henneberg, Poul L Christensen, Lars S Rasmussen

**Affiliations:** 1 Department of Anaesthesia, Centre of Head and Orthopaedics, Copenhagen University Hospital, Rigshospitalet, Blegdamsvej 9, DK-2100, Copenhagen Ø, Denmark; 2 Department of Anaesthesia, The Juliane Marie Centre, Copenhagen University Hospital, Rigshospitalet, Blegdamsvej 9, 2100, Copenhagen Ø, Denmark; 3 Department of Anaesthesia, Næstved Hospital, Ringstedgade 61, 4700, Næstved, Denmark

**Keywords:** Pulmonary gas exchange, Pulmonary atelectasis, Functional residual capacity, Pulmonary function, Anesthesia, General, Oxygen, Partial pressure, Surgery, Gynaecological

## Abstract

**Background:**

A high perioperative inspiratory oxygen fraction (FiO_2_) may reduce the frequency of surgical site infection. Perioperative atelectasis is caused by absorption, compression and reduced function of surfactant. It is well accepted, that ventilation with 100% oxygen for only a few minutes is associated with significant formation of atelectasis. However, it is still not clear if a longer period of 80% oxygen results in more atelectasis compared to a low FiO_2_.

Our aim was to assess if a high FiO_2_ is associated with impaired oxygenation and decreased pulmonary functional residual capacity (FRC).

**Methods:**

Thirty-five patients scheduled for laparotomy for ovarian cancer were randomized to receive either 30% oxygen (n = 15) or 80% oxygen (n = 20) during and for 2 h after surgery. The oxygenation index (PaO_2_/FiO_2_) was measured every 30 min during anesthesia and 90 min after extubation. FRC was measured the day before surgery and 2 h after extubation by a rebreathing method using the inert gas SF_6_.

**Results:**

Five min after intubation, the median PaO_2_/FiO_2_ was 69 kPa [53-71] in the 30%-group vs. 60 kPa [47-69] in the 80%-group (*P* = 0.25). At the end of anesthesia, the PaO_2_/FiO_2_ was 58 kPa [40-70] vs. 57 kPa [46-67] in the 30%- and 80%-group, respectively (*P* = 0.10). The median FRC was 1993 mL [1610-2240] vs. 1875 mL [1545-2048] at baseline and 1615 mL [1375-2318] vs. 1633 mL [1343-1948] postoperatively in the 30%- and 80%-group, respectively (*P* = 0.70).

**Conclusion:**

We found no significant difference in oxygenation index or functional residual capacity between patients given 80% and 30% oxygen for a period of approximately 5 hours.

**Trial registration:**

ClinicalTrials.gov Identifier: NCT00637936.

## Background

Atelectasis is a common perioperative complication [1]. It is observed in more than 90% of all anesthesitized patients with an average of 3-4% collapsed lung area and 10-15% collapsed lung tissue.

Main mechanisms underlying the atelectasis formation are compression, loss of surfactant or impared surfactant function and absorption of gas (oxygen) from alveoli behind closed or intermittently closed airways [2,3]. Ventilation for only a few minutes with 100% oxygen causes a significant increase in atelectasis shortly after anesthesia induction compared to ventilation with lower oxygen concentration [4]. However, no clear association between atelectasis formation and oxygen concentration during maintenance of anesthesia has yet been established [3], and a number of other harms and benefits still need further investigation [5].

A FiO_2_ of 0.80 during and for the first few h after elective colorectal surgery has been associated with a reduced frequency of surgical site infection [6,7]. It has been investigated in thirty patients how this high oxygen concentration for a prolonged time affects postoperative pulmonary function compared to a FiO_2_ of 0.30 [8]. That study found no significant changes in forced vital capacity (FVC), forced expiratory volume (FEV_1.0_), arterial oxygen tension (PaO_2_), alveolar-arterial oxygen difference ((Aa)DO_2_) or frequency and severity of atelectasis as measured first postoperative day. Atelectasis determined by computed tomography (CT) was seen in 94% of the patients given 0.80 FiO_2_ compared to 64% of patients given 0.30 FiO_2_ (*P* = 0.12).

Atelectasis causes a reduction in gas exchange capacity, and this can be estimated by measuring the functional residual capacity (FRC) [9]. Atelectasis leads to intrapulmonary right to left shunts [10], which can be assessed by a decrease in the ratio between the arterial oxygen tension and the inspired oxygen concentration, the oxygenation index (PaO_2_/FiO_2_) [11]. An increase in PaO_2_/FiO_2_ was found in patients given 0.40 FiO_2_ compared to patients given 1.0 FiO_2_ during laparoscopic cholecystectomy [12]. However, the perioperative changes in PaO_2_/FiO_2_ and the FRC have not yet been investigated in patients given 0.80 FiO_2_ or 0.30 FiO_2_.

The aim of this study was to compare the changes in perioperative PaO_2_/FiO_2_ and FRC in patients given 0.80 FiO_2_ or 0.30 FiO_2_ during and for 2 h after surgery for ovarian cancer. We hypothesized that a FiO_2_ of 0.80 would result in a larger reduction in PaO_2_/FiO_2_ at the end of anesthesia than a FiO_2_ of 0.30.

## Methods

Danish Medicines Agency and Local Ethics Committee approved the study (NCT00637936), and a written informed consent was obtained from all subjects.

The study was conducted from March 2008 to July 2008. Eligible patients were aged 18 years or older, scheduled for explorative laparotomy for ovarian cancer, defined as a Risk of Malignancy Index (RMI) ≥ 200 [13]. Exclusion criteria were: inability to give informed consent, inability to keep arterial oxygen saturation (SpO_2_) above 90% without supplemental oxygen, chemotherapy within 3 months, and surgery within 30 days (except surgery under local anesthesia or dilation and curettage under general anaesthesia). The study was a part of a multicenter trial investigating the influence of FiO_2_ on surgical site infection, the PROXI-trial [14,15]. However, all patients in this study were recruited in one centre.

Patients were randomized by a central interactive voice-response system at the Copenhagen Trial Unit to either FiO_2_ = 0.30 (30%-group) or FiO_2_ = 0.80 (80%-group) with stratification for center, diabetes mellitus, acute or elective surgery and body mass index (< 30 kg/m^2^ vs. ≥ 30 kg/m^2^).

All patients received paracetamol 1 g and diclofenac 50 mg orally preoperatively and pain intensity was monitored in the postanesthesia care unit using the Visual Analog Scale (VAS, scale 0–100). Analgesics were administered if VAS exceeded 30 using opioids, non-steroid anti-inflammatory drugs or local anesthesia epidurally if applicable.

Patients were preoxygenated with 100% oxygen for 5 min. Anesthesia was induced with intravenous (IV) administration of propofol 2 mg/kg together with fentanyl or remifentanil, and rocuronium 0.6 mg/kg was given to facilitate endotracheal intubation. Anesthesia was maintained with propofol or sevoflurane, and remifentanil.

After intubation, the lungs were ventilated with an adjusted fraction of inspired oxygen of 1.0 for 5 min until the first arterial blood sample was obtained. Subsequently, they received the allocated FiO_2_ until immediately before extubation, when an adjusted fraction of inspired oxygen of 1.0 was given.

The level of positive end-expiratory pressure (PEEP) was kept at 5 cmH_2_O. The lungs were ventilated by volume control ventilation with a tidal volume of 8–10 mL/kg and a respiratory frequency of 10–12 per min, aiming at an end-tidal carbon dioxide concentration of 4.5 to 6.0 kPa.

Fluid was given only to replace measured or calculated deficits (no third space loss) aiming at a body weight increase less than 1 kg [14]. Peroperative blood loss was replaced 1:1 with colloids, not exceeding 500 mL more than estimated blood loss [14]. Ephedrine, metaoxedrine or dopamine-infusion was used to keep the systolic arterial pressure > 90 mmHg. PEEP was primarily increased, if hypoxaemia was detected or suspected in order to keep the SpO_2_ above 94% and the PaO_2_ above 9 kPa. If this did not improve oxygenation, FiO_2_ was increased. Lung recruitment manoeuvres were not allowed in the period from preoxygenation to 2 h after extubation. Neuromuscular block was assessed in all patients using train-of-four (TOF) monitoring with the TOF-watch® (Danmeter APS, Odense, Denmark). Patients were extubated, when they were fully awake and TOF-ratio ≥ 0.90.

After arrival to the postanesthesia care unit, patients were given the allocated FiO_2_ for 2 h by a non-rebreathing facemask with a reservoir (High Concentration Oxygen Mask, Intersurgical Ltd, Wokingham, UK). Patients in the 30%-group were given a mixture of 2 litres of oxygen and 14 litres of air per minute and patients in the 80%-group were given a mixture of 14 litres of oxygen and 2 litres of air per minute. Subsequently, oxygen was given only at the physician’s discretion according to usual clinical practice, which in our institution will be to administer supplemental oxygen to keep SpO_2_ above 94% in a lung-healthy patient. The physician was blinded to group-allocation. Patients were monitored with continous pulse oximetry and no chest physical therapy was given in the postanesthesia care unit.

Arterial blood samples were obtained with 2-mL syringes containing heparin from a radial arterial catheter, transported in iced water and analysed with ABL System 777 blood gas analyser (Radiometer, Copenhagen, Denmark) within 10 min. Samples were obtained: five min after intubation, every half h during surgery, and 90 min after extubation. Five samples were drawn at each measurement and PaO_2_ was calculated as the average of three values after omitting the highest and the lowest values.

FRC was measured the day before surgery and 2 hours after extubation using the inert gas-rebreathing method and the Innocor-system (Innovision, Copenhagen, Denmark) [16,17]. The Innocor-system measured the change in the concentration of SF_6_ after 30 sec of rebreathing with the patient in the supine position and the upper body elevated 45°. The test was repeated and FRC calculated as the average of the two measurements. The quality of the data was evaluated on the basis of predefined criteria [18,19] by an investigator blinded to allocation and if of inadequate quality, they were excluded from further analysis.

Patients were seen daily by a study investigator, and were examined according to routine clinical practice by the attending physician, if she presented with symptoms of pulmonary complications, including chest radiographs or CT, when relevant. The radiologist was specifically instructed to evaluate the severity of atelectasis with Joyce et al. modification [20] of Wilcox severity scoring [21]: 0 = no atelectasis; 1 = plate atelectasis; 2 = segmental atelectasis; 3 = partial lobar atelectasis; 4 = complete atelectasis of one lung lobe; and 5 = complete atelectasis of one lung lobe in addition to any of the above.

To maintain blinding perioperative FiO_2_, flow of oxygen and air and PaO_2_ were collected on separate sheets and placed in sealed, opaque envelopes until data analysis. The patients, surgical staff, the radiologists and the specialist, who evaluated the FRC data, were not informed about group allocation.

The primary outcome was the change in PaO_2_/FiO_2_ at end of anesthesia. The secondary outcomes were change in PaO_2_/FiO_2_ 90 min after extubation, change in FRC 2 h after extubation, incidence and severity of atelectasis within 14 days, and SpO_2_ 2 h and 3 days after surgery.

Major violations to the protocol were defined as: lung recruitment manoeuvre, FiO_2_ above 0.60 in the 30%-group, PEEP ≥ 10 cmH_2_O for more than 1 h, and failure to use the oxygen mask for more than 1 h.

### Statistics

Data are reported with mean ± SD or median [interquartile range]. Data were compared with the Mann–Whitney test and categorical data with the *χ*^2^ test using SAS for Windows, version 9.1 (SAS Institute Inc., Cary, NC, USA). *P* < 0.05 was considered statistically significant.

We considered a 4 kPa difference in PaO_2_/FiO_2_ to be clinically relevant and estimated a SD of 4 kPa based on data from a previous study [12]. We calculated that a total sample size of 30 patients would allow us to detect this difference with a power of 80% and a significant level of 0.05.

## Results

**Figure 1:**
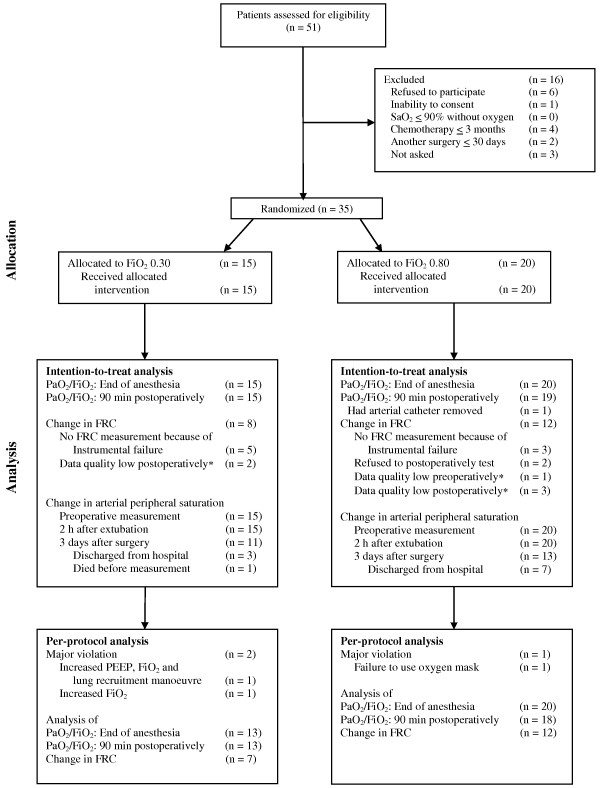
**Flow chart of patients undergoing surgery for ovarian cancer.** * Data quality evaluated as inadequate on the basis of predefined criteria by an investigator blinded to allocation [18,19].

We included 35 patients in this study (Figure 1, Table 1).

**Table 1:** Demographic and perioperative characteristics for 35 patients undergoing surgery for ovarian cancer

	**FiO**_**2**_ **= 0.30**	**FiO**_**2**_ **= 0.80**
	**(n = 15)**	**(n = 20)**
Age (years)	59 ± 16	64 ± 13
Body mass index (kg/m^2^)	23 ± 4	27 ± 8
Body weight (kg)	66 ± 15	72 ± 19
Smoking status		
Previous smoker	1 (8%)	3 (17%)
Current smoker	5 (33%)	6 (30%)
ASA physical status I/II/III	5/8/2	4/12/4
Co-existing diseases		
Hypertension	6 (40%)	12 (60%)
Other cardiovascular disease	1 (7%)	5 (25%)
Respiratory disease	1 (7%)	1 (5%)
Preoperative hemoglobin (mmol/l)		
	7.8 ± 0.7	7.6 ± 0.7
Amount of ascites		
< 50 mL	9 (60%)	13 (65%)
50-999 mL	3 (20%)	1 (5%)
1000-2999 mL	3 (20%)	1 (5%)
≥ 3000 mL	0 -	5 (25%)
Epidural analgesia	14 (93%)	14 (70%)
Morphine dose (mg)*	5 [0–10]	0 [0–6]
Duration of F_I_O_2_ = 1.0 (min)	14 [13–16]	15 [13–17]
Duration of anesthesia (min)	179 [140–282]	154 [118–256]
Blood loss (mL)	600 [355–1200]	575 [75–1385]
Crystalloid infused (mL)	2000 [1220–2500]	1735 [1160–3500]
Colloid infused (mL)	500 [0–1000]	500 [500–1020]
Patients receiving blood	6 (40%)	5 (25%)
Units of blood transfused	2 ± 1	4 ± 2
Ephedrine dose (mg)	30 [0–30]	30 [10–48]
Other vasopressors	7 (47%)	8 (40%)

The data are presented as mean ± SD, number of patients (percentage) or median [interquartile range].

FiO_2_ = inspired oxygen fraction; ASA = American Society of Anesthesiologists.

* Total morphine dose received 2 h postoperatively, including equivalent doses of fentanyl.

Five min after intubation, the median PaO_2_/FiO_2_ was 69 kPa [53-71] in the 30%-group and 60 kPa [47-69] in the 80%-group (*P* = 0.25). At end of anesthesia, the PaO_2_/FiO_2_ was 58 kPa [40-70] vs. 57 kPa [46-67] in the 30%- and 80%-group, respectively (*P* = 0.10, Table 2). Ninety min after extubation, the PaO_2_/FiO_2_ was reduced to 56 kPa [37-60] in the 30%-group and 50 kPa [42-57] in the 80%-group (*P* = 0.66).

**Table 2:** Pulmonary function in 35 patients undergoing surgery for ovarian cancer

	**FiO**_**2**_ **= 0.30**	**FiO**_**2**_ **= 0.80**	***P*value**
	**(n = 15)**	**(n = 20)**	
PaO_2_/FiO_2_ (kPa)			
5 min after intubation *	69 [53–71]	60 [47–69]	0.25
30 min after intubation	58 [37–72]	58 [44–67]	0.39
End of anesthesia	58 [40–70]	57 [46–67]	0.10
90 min after extubation	56 [37–60]	50 [42–57]	0.66
FRC (mL) #			
Preoperative	1993 [1610–2240]	1875 [1545–2048]	
2 h after extubation	1615 [1375–2318]	1633 [1343–1948]	0.70
Arterial oxygen saturation			
Preoperative (%) §	97 ± 2	96 ± 2	
≤ 95% 2 h after extubation §	3 (20%)	6 (33%)	0.50
≤ 95% 3 days after surgery §	2 (13%)	7 (35%)	0.15
Atelectasis ‡			
No atelectasis	13 (87%)	15 (75%)	0.51
Plate atelectasis	0	0	
Segmental atelectasis	1 (7%)	0	
Partial lobar atelectasis	0	0	
Complete atelectasis of one			
lung lobe	0	2 (10%)	
Complete atelectasis of one			
lung lobe in addition to any of			
the above	1 (7%)	3 (15%)	

The data are presented as mean ± SD, number of patients (percentage) or median [interquartile range].

*P* values are calculated by Mann–Whitney test to compare the changes from baseline values between the two groups.

P_a_O_2_ = arterial oxygen tension; FiO_2_ = inspired oxygen fraction; PaO_2_/FiO_2_ = oxygenation index;

FRC = functional residual capacity.

* Measured at FiO_2_ =1.0.

# Preoperative data were missing in 9 patients. Postoperative data were missing in 15 patients (Figure 1).

§ Measured with the patient breathing room air.

‡ Severity of atelectasis assessed according to Joyce et al. modification [20] of Wilcox severity scoring [21].

**Figure 2:**
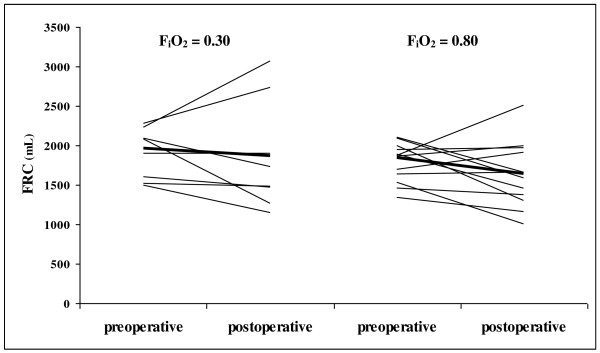
**Mean changes in functional residual capacity (FRC) in 35 patients undergoing surgery for ovarian cancer.** Solid line represents the group’s mean. FiO_2_ = inspired oxygen fraction. Data were missing in 15 patients because of instrumental failure (5 and 3 patients in the 30%- and 80%-group, respectively), inadequate data quality (2 and 3 patients) and 2 patients in the 80%-group refused the postoperative test (Figure 1).

Data on FRC with adequate quality were collected in 20 patients (Figure 1). The median FRC were 1993 mL [1610-2240] vs. 1875 mL [1545-2048] at baseline and 1615 mL [1375-2318] vs. 1633 mL [1343-1948] postoperatively in the 30%- and 80%-group, respectively (*P* = 0.70) (Table 2, Figure 2).

Changes in PaO_2_/FiO_2_ and FRC are reported in Table 3. No significant difference was found between the groups.

**Table 3:** Changes in pulmonary function in 35 patients undergoing surgery for ovarian cancer.

	**Change in PaO**_**2**_**/FiO**_**2**_**at the end of anesthesia (kPa)**			**Change in FRC 2 h after extubation (mL)**		
	**FiO**_**2**_** = 0.30**	**FiO**_**2**_** = 0.80**	***P*value**	**FiO**_**2**_** = 0.30**	**FiO**_**2**_** = 0.80**	***P*value**
	**(n = 15)**	**(n = 20)**		**(n = 8)**	**(N = 12)**	
Intention-to-treat	−7.5 ± 10	-1.2 ± 10	0.10	-49 ± 507	−152 ± 382	0.70
	n = 13	n = 20		n = 7	n = 12	
Per-protocol *	−7.1 ± 9	-1.2 ± 10	0.10	60 ± 435	−152 ± 382	0.35

The data are mean difference form baseline ± SD. *P* values are calculated by Mann–Whitney test to compare the changes from baseline values between the two groups. PaO_2_ = arterial oxygen tension; FiO_2_ = inspired oxygen fraction; PaO_2_/FiO_2_ = oxygenation index; FRC = functional residual capacitity.

* Excluded patients are described in Figure 1.

Five patients (25%) in the 80%-group developed radiologically verified atelectasis compared to 2 patients (13%) in the 30%-group (*P* = 0.51), and there were no significant differences in postoperative SpO_2_ (Table 2).

Three patients had major protocol violations (Figure 1), but the exclusion of these patients did not change the results significantly (Table 3). In order to keep PaO_2_ above 9 kPa additionally one patient in the 30%-group received 58% oxygen for one hour and one patient in the 80%-group had PEEP adjusted to 7 cmH_2_O. In the postanesthesia care unit one patient in the 30%-group was given the allocated mixture of 2 litres of oxygen and 14 litres of air per minute until the last arterial blood sample had been drawn. Thereafter, the patient received a mixture of 4 litres of oxygen and 12 litres of air per minute until measurement of FRC and SpO_2_. SpO_2_ was measured with the patient breathing air.

A post hoc analysis of the alveolar-arteriel oxygen difference (AaO_2_ gradient) showed no significant baseline difference between the two groups. Mean AaO_2_ gradient 5 min after intubation was 31 ± 16 kPa in the 80%-group and 27 ± 15 kPa in the 30%-group (*P* = 0.31).

## Discussion

Contrary to our primary hypothesis, we did not find a significant difference in PaO_2_/FiO_2_ at end of anesthesia between patients given a high perioperative oxygen fraction of 80% and 30%. Moreover, we did not find a significant difference in FRC or in the calculated changes.

The accuracy of the measurements was high because five blood samples were obtained in rapid succession at each PaO_2_ measurement and at established FiO_2_-levels [22]. Moreover, FRC were calculated as an average of two measurements both pre- and postoperatively and only data of adequate quality were included.

Although we used stratified randomization, some baseline characteristics showed a trend towards imbalance. The 80%-group seemed to be at higher risk of pulmonary complications based on age, body mass index, amount of ascites, and the proportions of previous smokers and cardiovascular diseases [23-25]. However, there was no significant difference in baseline value of PaO_2_/FiO_2_ or AaO_2_ gradient between the two groups. It is a possible limitation that removal of ascites could result in an improvement of PaO_2_/FiO_2_ and FRC, but we found a similar change in pulmonary function in the five patients (all in the 80%-group) with more than 3000 mL of ascites as we found in the remaining patients.

We used the body mass index to assess obesity status, but a detailed evaluation of body fat distribution (e.g. waist-to-hip ratio) might be a more accurate predictor of pulmonary complications [26]. Anyhow, body mass index is easier to determine and is consistent with most of the previous studies evaluating perioperative FiO_2_.

All patients were preoxygenated with 100% oxygen and received 100% oxygen just before extubation. Completely avoiding a high FiO_2_ in the 30%-group would have been an advantage regarding atelectasis formation, but a lower FiO_2_ than 1.0 during preoxygenation and immediately prior to tracheal extubation is generally not recommended, because it reduces the margin of safety significantly [4], considering the risk of difficult airway management. In this study, ventilation with 100% oxygen after intubation was additionally used for a relatively long period in order to ensure baseline equilibrium of PaO_2_ and FiO_2_. This method of increasing baseline reliability may on the other hand have contributed to further atelectasis formation [4]. It is possible that the amount of atelectasis was similar in both groups immediately after preoxygenation and induction of anesthesia and remained unchanged hereafter irrespective of the subsequent FiO_2_, which is a potential limitation of this study [4].

The change in the PaO_2_/FiO_2_ is not a perfect measurement to quantify the amount of impaired gas exchange. It is affected by numerous factors other than true intrapulmonary right to left shunts caused by atelectasis, such as changes in circulatory blood volume, vasopressor use, positioning of the patient and body temperature. These factors were, however, not different between the groups. Other factors such as ventilation-perfusion (V_A_/Q) inequality and hypoxic pulmonary vasoconstriction can also have modified the PaO_2_/FiO_2_. Moreover the PaO_2_/FiO_2_ is not linearly correlated to FiO_2_[22,27]. As outcome we measured the change in the PaO_2_/FiO_2_ from samples drawn at two different FiO_2_-levels in each patient: baseline at FiO_2_ 1.0 and endpoints at FiO_2_ 0.3 or 0.8. However, the mean difference between the groups in PaO_2_/FiO_2_ change from 30 min after intubation to the end of anesthesia (measured at the same FiO_2_-level in each group) was only 3.0 kPa (*P* = 0.39). We therefore consider it most likely that any difference in PaO_2_/FiO_2_ must be related to the induction of anesthesia including the change from 1.0 to the allocated FiO_2_.

The FRC measurements required patients to breathe deeply for 30 sec with a respiratory frequency of 20 per min. The 7 patients (5 in the 80%-group) who were unable to cooperate sufficiently or declined to perform the test, had a longer duration of anesthesia, a larger blood loss, and also a larger reduction in PaO_2_/FiO_2_. Thus, an effect of high FiO_2_ on the change in FRC could have been overlooked, because FRC data in these patients were missing. The need for cooperation is a disadvantage of the inert gas-rebreathing method in the clinical setting even though it can be very useful in healthy individuals or mechanically ventilated patients.

The mean difference in PaO_2_/FiO_2_ reduction between the 30%-group and the 80%-group was 6.3 ± 10 kPa. Therefore, we cannot exclude that a clinically important difference exists, but our SD was larger than expected and it would require almost 200 patients to detect this difference.

The application of PEEP = 5 cmH_2_O may have reduced the amount of intraoperative atelectasis and improved the oxygenation [28], however PEEP was applied equally to all patients exept for 1 in the 30%-group. Hedenstierna et al. showed in a study of 12 patients a trend towards reoccurence of atelectasis within 5 min after discontinuation of PEEP [29] In contrast, a Cochrane review from 2010 indicated that intraoperative PEEP of 5–10 cm H_2_O may reduce postoperative atelectasis and improve postoperative gasexchange (PaO_2_/FiO_2_) [30].

We found a mean reduction in FRC of 8%, and this is less than the 20% reduction measured shortly after intubation [31], but in accordance with the 12% reduction found on the first day after lower abdominal surgery [32].

A postoperative reduction in FRC may not solely be caused by collapse of some lung units (atelectasis), but also by a general change in intrathoracic volumes caused by reflex diaphragmatic dysfunction, shallow breathing, mechanical disruption of the abdomen, pulmonary edema, increased abdominal blood volume or postoperative incisional pain[32,33]. We found that the change in FRC and in postoperative PaO_2_/FiO_2_ tended to be larger in the patients, who did not receive thoracic epidural analgesia and this was the case for 6 of the 20 patients in the 80%-group and 1 of the 15 patients in the 30%-group. However, none of these patients developed radiologically verified atelectasis. Epidural analgesia improves some aspects of pulmonary function, which may be attributed to better pain control [34]. We used standardized pain relief to ensure that all patients had adequate analgesia even if epidural was not used. We found no significant difference in overall opiod consumption between the groups (Table 1).

Our observed changes in FRC may have been too small to affect the PaO_2_/FiO_2_ as noted by Dueck et al. [35]. Their study showed only a small effect of FRC reduction on the degree of pulmonary shunt until FRC/TLC was less than 0.30, i.e. less than awake, supine closing capacity.

Akca et al. found no significant difference in incidence of atelectasis assessed by CT (64% vs. 94%; *P* = 0.12) in patients given 30% or 80% oxygen, respectively [8]. Such high incidences are related to the high sensitivity of a CT-scan. In our study, seven patients (20%) developed radiological verified atelectasis in addition to pulmonary symptoms. With this definition of atelectasis, no significant difference in atelectasis was found among the 1,400 patients included in the PROXI-trial (7.9% vs. 7.1% in the 80% and 30% oxygen group, respectively (*P* = 0.60)) [15]. Edmark et al. showed that the benefit of using 80% oxygen compared to 100% oxygen during induction of anesthesia in order to reduce atelectasis diminished gradually with time [36]. These results may indicate that other factors than the oxygen concentration are important to the formation of late peroperative atelectasis. Moreover, patients who are treated with hyperbaric oxygen usually receive oxygen at a pressure of 240 kPa in 3 cycles of 30 min show no clinically significant signs of reduction in pulmonary function [37]. This is in accordance with the results found in our study

The patients included were middle-aged women, undergoing explorative laparotomy of a substantial duration. We cannot reject the possibility that 80% oxygen for a prolonged period may affect PaO_2_/FiO_2_ and FRC more in heavy smokers or among patients with severe pulmonary or cardiovascular comorbidity.

## Conclusion

Ventilation with 80% oxygen for several hours was not significantly associated with changes in the perioperative oxygenation index or functional residual capacity compared to ventilation with 30% oxygen. However, a larger trial is required to exclude that an important difference in oxygenation or other clinical outcomes exists.

## Competing interests

The authors have no financial or non-financial competing interests to declare. This study has no industrial funding or support.

## Authors’ contributions

AKS is the prinicpal investigator of the study and was the primary author of the manuscript. CSM, PLC, SH and LSR were all involved in study design and made contributions to the manuscript. All authors have read and approved the final version.
